# The burden of typhoid fever in Klang Valley, Malaysia, 2011–2015

**DOI:** 10.1186/s12879-020-05500-x

**Published:** 2020-11-16

**Authors:** Eida Nurhadzira Muhammad, Mohd Hatta Abdul Mutalip, Mohd Hazrin Hasim, Faizah Paiwai, Sayan Pan, Mohd Amierul Fikri Mahmud, Norzawati Yeop, Guat Hiong Tee, A’ Aishah Senin, Tahir Aris

**Affiliations:** 1 Center of Communicable Disease Research, Institute for Public Health, National Institutes of Health, Ministry of Health, Shah Alam, Selangor Malaysia; 2 Pathology Department, Hospital Tawau, Ministry of Health Malaysia, Tawau, Sabah Malaysia; 3 Food Safety and Quality Division, Perlis Health State Department, Ministry of Health Malaysia, Kangar, Perlis Malaysia; 4 University Malaya, Kuala Lumpur, Malaysia; 5 Sector of Vaccine Prevention/Food and Water Borne Diseases, Disease Control Division, Ministry of Health Malaysia, Putrajaya, Malaysia

**Keywords:** Typhoid fever, Klang Valley, MDR *Salmonella typhi*

## Abstract

**Background:**

Typhoid fever causes global morbidity and mortality and is a significant health burden, particularly in low- and middle-income countries. The direct fecal-oral route is the main transmission mode, but indirect environmental transmission could occur, particularly in urban settings. This study aimed to investigate the burden and trend of typhoid fever, reporting the coverage system between government and private practice and pattern of multidrug-resistant (MDR) typhoid cases in the urban Klang Valley area from 2011 to 2015.

**Methods:**

The data from a cross-sectional study retrieved from the e-Notifikasi System, a national reporting system for communicable diseases provided by the Disease Control Division, Ministry of Health Malaysia and secondary data of all the typhoid cases were obtained from the public and private hospitals and laboratories in Klang Valley. Descriptive analysis was performed to examine the sociodemographic characteristics, spatial mapping was conducted to examine trends, and the crude incidence rates of confirmed typhoid cases and percentage of reporting coverage were calculated. Significant differences between MDR and non-MDR *Salmonella typhi* were determined in the patient’s sociodemographic characteristics, which were analyzed using χ^2^ test. *P* values < 0.05 were considered statistically significant.

**Results:**

In total, 507 typhoid fever cases were reported in Klang Valley; however, only 265 cases were confirmed by culture tests. The crude incidence rates of confirmed cases were between 0.5 to 0.7 but peaked at 1.42 per 100,000 population in 2015. Most typhoid fever cases were observed among men (55.6%), individuals aged 21 to 30 years (27.6%), Malaysians (86.3%) and individuals of Malay ethnicity (52.1%). The reporting coverage of confirmed cases was 78.9% and non-reporting coverage of unconfirmed typhoid cases was 79.5%. The predictive value positive (PVP) was 89.3, and 7.5% were detected as MDR *Salmonella typhi*. Statistical significance was found in gender, citizenship and ethnicity regarding MDR *Salmonella typhi* (*p* = 0.004, *p* = 0.008 and *p* = 0.034, respectively).

**Conclusions:**

The local transmission of typhoid is still prevalent in the Klang Valley despite rapid urbanization and development in recent years. These findings are essential for policy makers to plan and implement focused and effective preventative activities to curb typhoid infection in urban areas.

## Background

Typhoid is a contagious enteric infection associated with the ingestion of food or drinks contaminated by the bacterium *Salmonella enterica serovar typhi*. Typhoid causes global morbidity and mortality and is a significant health burden, particularly in low- and middle-income countries. Global trends of typhoid fever reported by the Institute for Health Metrics have shown a steady decline in the disability-adjusted life years (DALYs) from 280.77 per 100,000 population in 1990 to 110.43 per 100,000 population in 2017 [1]. However, an estimate from the Global Burden of Disease Study in 2017 using modeling methods reported the incidence is still high as 11 million typhoid cases were reported in 2017 with more than 116,000 deaths attributable to this disease [2]. Surveillance data from five Asian countries in urban and urban slum areas with the targeted group have shown 81.7 and 493.5 cases per 100,000 population per year in Indonesia and India, respectively, at all age groups, 451.7 cases per 100,000 per year from age 2 to 15 years in Pakistan, 24.2 cases per 100,000 per year in people aged 6 to 18 years in Vietnam and 15.3 cases per 100,000 per year in people aged 5 to 60 years in China [3]. Heavily populated areas with poor conditions of the house and neighborhood were observed to be risks of typhoid in many urban slum or rural areas.

A recent report on typhoid fever in Malaysia noted a low incidence of 0.59 and a death rate of 0.02 per 100,000 population [4]. A review on diarrheal diseases also reported a decreased pattern of typhoid cases in Malaysia over the years [5]. However, typhoid is more prevalent in certain states where it is still endemic in the east coast of Malaysia [4, 6]. It is more common in rural areas where limited access to clean water and poor sanitation increases the risk of contracting typhoid infection. Infection is also contributed by unhygienic food practices among street hawkers who usually do not comply with the safe and healthy procedural guidelines [6, 7]. Despite being a rural disease, a recent increase in the incidence of typhoid in urban areas in Malaysia has sparked public health concern. Numerous preventative efforts, including regulations and enforcement of food hygiene and safety have been implemented, but sporadic and clusters of typhoid cases are still being reported in urban areas.

Poor management in the diagnosis and non-compliance of antibiotic use contribute to the spread of typhoid infection that subsequently could increase the cases of drug-resistant typhoid [2]. Globally, the reported cases of multidrug-resistant (MDR) typhoid are on increasing. The emergence of MDR typhoid is a threat to global public health, and this situation will result in higher antibiotic concentration use, increasing the cost of treatment. The slow development of new antibiotics treatment for typhoid could further exacerbate the threat from drug-resistant typhoid. Hence, this study aimed to investigate the burden and trend of typhoid fever in the urban Klang Valley area over 5 years from 2011 to 2015. We would also like to examine the reporting coverage system of typhoid cases between the government and private practices and the pattern of MDR typhoid cases that could potentially have caused a major prolonged outbreak in the Klang Valley area within the five-year period.

## Methods

### Study area and population

In this study, we chose urban localities in the Klang Valley area, located in Kuala Lumpur and Selangor, Malaysia. For the state of Selangor, the following four districts were included in this study: Petaling, Klang, Gombak and Hulu Langat. For the state of Kuala Lumpur, we included all areas. These areas are the most populous in Malaysia, with an estimated population of 6,373,500 in 2011 to 6,924,600 in 2015.

### Source of data and study design

A cross-sectional study involving data analysis of all notified typhoid cases in the selected study localities were included in this study. We retrieved data from the e-Notifikasi System, a national reporting system for communicable diseases provided by the Disease Control Division, Ministry of Health Malaysia. All the positive typhoid cases from the public and private hospitals and laboratories must notify the Ministry of Health Malaysia, and all data will be recorded in the e-Notifikasi system.

We had also obtained secondary data, microbiological data of all typhoid cases from public and private hospitals and private laboratories in Klang Valley from year 2011 to 2015. Both datasets from the e-Notifikasi system and data sought from all health facilities were merged into one main dataset. The data that were not captured in the e-Notifikasi system but were obtained from the private health facilities including private laboratories were considered missing or not recorded in the e-Notifikasi system. We included all cases, whether confirmed, probable or suspected, who lived in the Klang Valley area and those reported with a positive *Salmonella typhi* test. Cases outside of the Klang Valley area were excluded from this study. This study was approved by the Medical Research and Ethics Committee (MREC), Ministry of Health Malaysia, with a registration number of NMRR-16-2042-31,954.

### Case definition

We used a standardized case definition for typhoid fever based on the infectious disease guidelines from the Ministry of Health, Malaysia [8]. Typhoid fever cases were defined as an illness with an insidious onset of prolonged fever, constitutional symptoms (e.g., malaise, headache, and anorexia), non-productive cough in the early stage of the illness, constipation more often than diarrhea and hepatosplenomegaly. Rose spots were often observed in fair-skinned patients. All cases of isolated *Salmonella typhi* from blood, stool or other clinical specimens were confirmed according to laboratory criteria. For case classification, the definitions were as follows:

- Suspected case: a case that fulfills the clinical case definition.
- Probable case: a suspected case with a positive serology or antigen detection test but without the isolation of *Salmonella typhi*
- Confirmed case: a suspected case with the isolation of *Salmonella typhi* from the blood, stool or other clinical specimens.

The management of outbreaks by the Ministry of Health, Malaysia defines outbreaks as when the disease occurs almost 2 times or more at a time incubation within the same locality or related to the linked epidemiology. The outbreak is declared to expire every new time within 42 days (2 incubations) from the start date of the last case [6]. In this study, the typhoid test performed by culture was defined as confirmed cases, while other tests such as Typhidot and Widal Weil Felix (WWF) were considered as non-confirmed cases. Reported cases were defined as cases where notification was conducted and were keyed into the e-Notifikasi system. Cases that were not reported were typhoid cases that were not keyed into the e-Notifikasi system.

### Microbiological and susceptibility tests

Laboratory testing is one of the important elements in healthcare management as evidence of suspected disease. Various tests were used depending on the current practices or availability of equipment at each hospital and laboratory:

- Culture and sensitivity test
  This test is used to isolate the bacteria on a specific medium from samples such as blood, stool and urine. If growth on the medium is detected, identification of the bacteria will be conducted. The sensitivity test, also called susceptibility testing, is the test to determine the ‘sensitivity’ of the bacteria toward the drug (antibiotic) used for treatment purposes. The drug panel tested was based on the bacteria isolated. In this study, for *Salmonella typhi*, the drugs used were chloramphenicol, co-trimoxazole, ampicillin, ciprofloxacin, ceftriaxone, tetracycline and nalidixic acid (CLSI Standardized antibiotic panel 2010).
- Typhidot test
  The Typhidot test is simple, rapid, and easy to perform, and no additional sample preparation is required. Using the principle of antigen-antibody complexes, the Typhidot kit will detect IgM and IgG antibodies against the specific outer membrane protein (OMP) antigen of the *Salmonella typhi* in human whole blood, serum or plasma [9]. The Typhidot result can be positive within 2 to 3 days after infection. The sensitivity of the Typhidot test is in the range of 66–88%, and the specificity of the Typhidot test is 75–91% [10].
- Widal Weil Felix (WWF) test
  Another test used for typhoid fever is the WWF test, which is an agglutination test that detects the presence of H and O antigens of *Salmonella typhi* in the patient’s serum. Patients with typhoid develop antibodies in their serum that can react with H and O antigens (in the reagent) to produce clumping on the test card and agglutination in the test tube. However, the WWF test has limitations. The test may generate false-positive results in patients who had previous immunization or *Salmonella typhi* infection. In addition to cross-reactivity with non-typhoid *Salmonella*, the test cannot distinguish between a current infection and a previous infection. In endemic areas, the existing baseline antibody level and repeated exposure to *Salmonella* infection in the population can interfere with the interpretation of the WWF titer unless these baseline values are known. False-negative results may occur when the previous antimicrobial treatment inhibits the antibody response [11]. In procedure preparation, a negative result can occur if an inadequate inoculum of bacterial antigen in the host was not sufficient to induce antibody production [12]. *Salmonella* antibody starts appearing in the serum at the end of the first week and rises sharply during the 3rd week of fever. In acute typhoid fever, O antigens can usually be detected 6–8 days after the onset of fever and H antigens after 10–12 days.

### Statistical analysis

Descriptive analysis was performed to examine the sociodemographic characteristics of typhoid fever cases in the Klang Valley area using SPSS Version 23. Typhoid fever cases were spatially mapped to examine the trends and clusters of typhoid fever cases in 5 years. We calculated the crude incidence rates of confirmed typhoid fever cases in the Klang Valley for 5 years. We also evaluated the coverage of reporting of typhoid cases from the laboratory, private practice and government health care facilities compared with the cases from the e-Notifikasi system. From all the total typhoid cases, the percentage of the reporting coverage was counted and the positive predictive value was defined as the percentage of confirmed cases among all the reported cases [13]. The significant difference between MDR and non-MDR *Salmonella typhi* was determined by the patient’s sociodemographic characteristics, which were analyzed using χ^2^ test. *P* values < 0.05 were considered statistically significant.

## Results

### Cases of *Salmonella typhi*

**Fig. 1:**
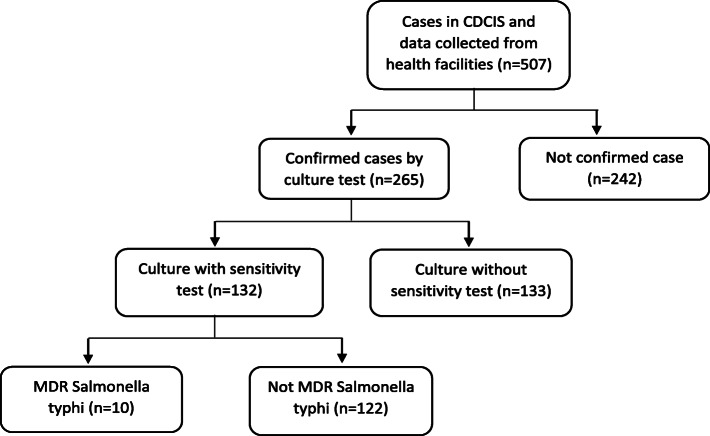
Flow of the typhoid cases in Klang Valley, Kuala Lumpur

**Fig. 2:**
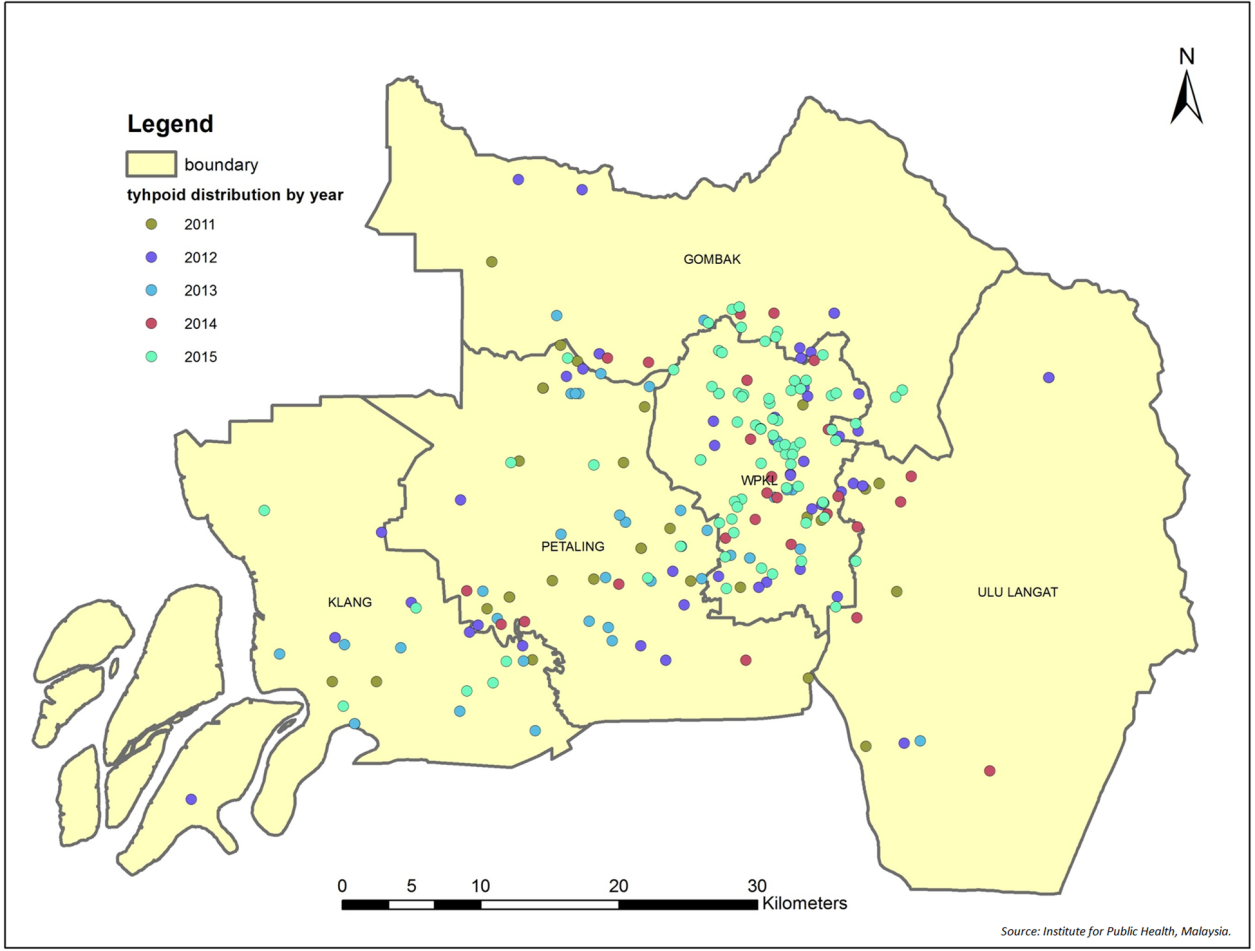
Study area and distribution of typhoid fever in Klang Valley, Kuala Lumpur from 2011 to 2015

In total, 507 cumulative typhoid fever cases were identified in the Klang Valley from 2011 to 2015, where 265 cases were confirmed while 242 cases were probable and suspected. The flow of cases and distribution of typhoid fever cases in the Klang Valley from 2011 to 2015 are illustrated in Figs. 1 and 2, respectively.

**Fig. 3:**
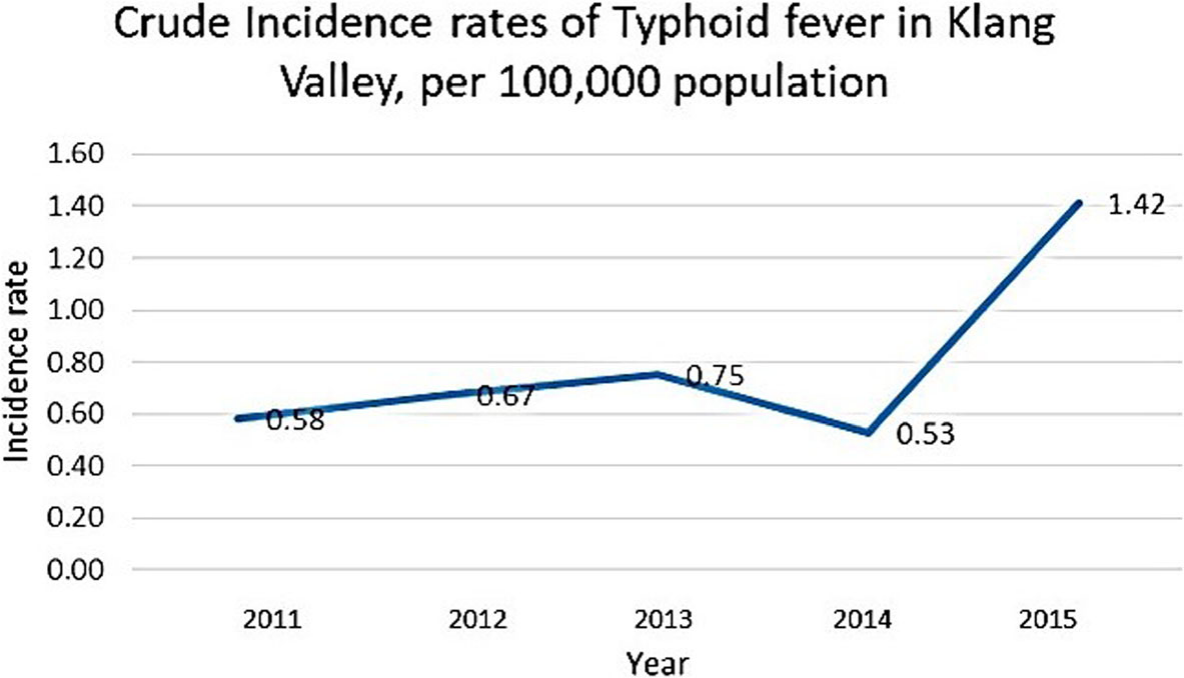
Incidence rates of typhoid fever in Klang valley for 5 years between 2011 and 2015 per 100,000 population

The incidence rates of confirmed typhoid fever cases showed an increasing trend over the past 5 years from 2011 to 2015. The incidence rate was between 0.5 to 0.7 and peaked at 1.42 in 2015 (Fig. 3). Within 5 years, the number of typhoid cases was reportedly the highest in the Gombak district (*n* = 34, 82.9%), followed by the Hulu Langat district (*n* = 33, 66.0%), Kuala Lumpur (*n* = 95, 65.5%), and Petaling (*n* = 79, 53.4%), and the Klang district was the lowest (*n* = 27, 22%).

The sociodemographic characteristics of the typhoid cases in Klang Valley are described in Table 1. Cases were more prevalent among males (55.6%), those aged 21 to 30 years (27.6%), Malaysians (86.3%) and those of Malay ethnicity (52.1%).

**Table 1:** Sociodemographic characteristics of typhoid cases in Klang Valley, 2011 to 2015, *n* = 507

Variable	Count	Percentage (%)
**Gender**		
Male	272	55.6
Female	217	44.4
^a^Missing	18	
**Age**		
0 to 10 years	64	14.3
11 to 20 years	73	16.4
21 to 30 years	123	27.6
31 to 40 years	78	17.5
41 to 50 years	49	11
> 51 years	59	13.2
^a^Missing	61	
**Citizenship**		
Malaysian	437	86.3
Non-Malaysian	70	13.7
**Ethnicity**		
Malay	264	52.1
Chinese	97	19.1
Indian	69	13.6
Bumiputra Sabah Sarawak	4	0.8
Others (foreigner)	73	14.4

^a^The data were not available in the e-Notifikasi system

### Laboratory test and reporting coverage

Among 507 typhoid cases, 265 cases were confirmed by culture, 122 cases by Typhidot and 120 cases by WWF. However, after consideration, the cases detected using the WWF test were removed from the data analysis because the test has many limitations and is not reliable. The reporting coverage of typhoid cases by diagnosis test and health facility (government or private hospital) are shown in Table 2. The reporting coverage of confirmed cases was 78.9%, and the non-reporting coverage on unconfirmed typhoid cases was 79.5%. The positive predictive value (PVP) was calculated as 89.3%.

**Table 2:** Reporting coverage of typhoid cases by hospital and final diagnosis, Klang Valley, Malaysia (*n* = 387)

Diagnosis	^a^Reported (n, %)		n, %	^a^Not Reported (n, %)		n, %
	Government hospital	Private hospital		Government hospital	Private hospital	
Confirmed with culture test	67.0 (*n* = 140)	33.0 (*n* = 69)	100 (*n* = 209)	78.6 (*n* = 44)	21.4 (*n* = 12)	100 (*n* = 56)
Not Confirmed with culture test (Typhidot test)	48.0 (*n* = 12)	52.0 (*n* = 13)	100 (*n* = 25)	0 (*n* = 0)	100 (*n* = 97)	100 (*n* = 97)
	65.0 (*n* = 152)	35.0 (*n* = 82)	100 (*n* = 234)	28.8 (*n* = 44)	71.2 (*n* = 109)	100 (*n* = 153)

^a^Definition as in the methodology section (case definition)

The overall reporting coverage was calculated as follows [13]:

- Reporting coverage of confirmed cases = (209 / 265) × 100 = 78.9%
- Non-reporting coverage of unconfirmed cases = (97 / 122) × 100 = 79.5%
- Predictive Value Positive (PVP) = (209 / 234) × 100 = 89.3%

### Antimicrobial resistance in *Salmonella typhi*

From 265 confirmed cases by culture test, only 132 had the drug sensitivity test result. The antibiotics tested in this analysis were ampicillin, chloramphenicol, ciprofloxacin, ceftriaxone, nalidixic acid, co-trimoxazole and tetracycline but not all cases were tested with all seven antibiotics. Nalidixic acid showed the highest resistance (36%), followed by ampicillin (11.1%) and co-trimoxazole (9.8%). The overall percentages of susceptibility, intermediate and resistance of antibiotics are described in Table 3.

**Table 3:** Overall percentage of susceptibility, intermediate and resistance of antibiotics, *n* = 132

Testing	Susceptibility		Intermediate		Resistant	
	n	%	n	%	n	%
Ampicillin	111	88.1	1	0.8	14	11.1
Chloramphenicol	95	90.5	0	0.0	10	9.5
Ciprofloxacin	94	75.8	22	17.7	8	6.5
Ceftriaxone	122	98.4	0	0.0	2	1.6
Nalidixic acid	14	56	2	8.0	9	36
Co-Trimoxazole	101	90.2	0	0.0	11	9.8
Tetracycline	34	91.9	0	0.0	3	8.1

**Fig. 4:**
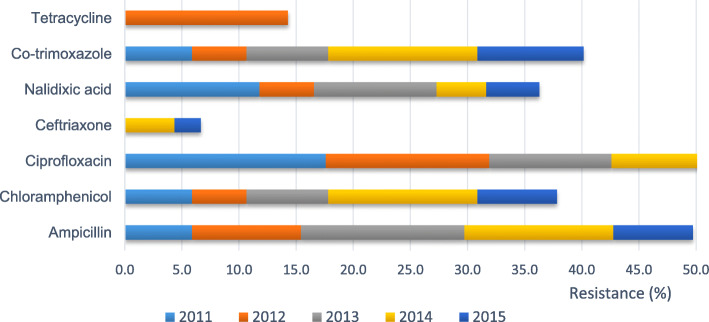
Trend of antimicrobial resistance to *Salmonella typhi* between 2011 and 2015 in Klang Valley, Kuala Lumpur. The graphs show the percentages of tetracycline, co-trimoxazole, nalidixic acid, ciprofloxacin, ceftriaxone, chloramphenicol and ampicillin resistance to *Salmonella typhi* by year

The trend of the antimicrobial resistance of *Salmonella typhi* over the 5 years in the Klang Valley showed both increased and decreased patterns according to the antibiotic (Fig. 4). The resistance of *Salmonella typhi* to ampicillin was 9.5% in 2012, increased to 14.3% in 2013, slightly decreased in 2014 (13.0%) and further decreased to 7.0% in 2015. The resistance to chloramphenicol and co-trimoxazole was 5.9% in 2011, with an increasing trend from 2012 to 2014 (4.8 to 13.0%) and decreasing to 7.0% (chloramphenicol) and 9.3% (co-trimoxazole) in 2015. For nalidixic acid, the resistance fluctuated at 11.8% (2011), 4.8% (2012), 10.7% (2013), 4.3% (2014) and 4.7% (2015). Ciprofloxacin showed a decrease pattern from 2011 to 2015 (17.6, 14.3, 10.7, 8.7 and 0.0%, respectively). Antimicrobial resistance to tetracycline was only observed in 2012 (14.3%), and resistance to ceftriaxone only in 2014 (4.3%) and 2015 (2.3%).

Multidrug resistance (MDR) to *Salmonella typhi* was detected in 7.5% of cases (*n* = 10), one MDR *Salmonella typhi* case each in 2011 and 2012, two cases in 2013 and three cases each in 2014 and 2015. MDR was considered if the isolates were resistant to three or more antimicrobial classes (ampicillin, chloramphenicol and co-trimoxazole) [14–16]. Statistically significant differences in MDR *Salmonella typhi* were observed according to gender (*p* = 0.004), citizenship (*p* = 0.008) and ethnicity (*p* = 0.034), but no significance difference was seen according to age group (*p* = 0.735) (Table 4).

**Table 4:** MDR *Salmonella typhi* percentages for gender, age, citizenship and ethnicity (*n* = 132)

	Criteria	MDR ***Salmonella typhi***		***p***-value
		Yes	No	
Gender	Male	10 (13.5%)	64 (86.5%)	0.004
	Female	0 (0%)	57 (100%)	
Age	≤ 10 years	0 (0%)	25 (100%)	0.735
	11–20 years	1 (5.6%)	17 (94.4%)	
	21–30 years	2 (7.1%)	26 (92.9%)	
	31–40 years	1 (5.9%)	16 (94.1%)	
	31–50 years	0 (0%)	9 (100%)	
	> 50	1 (10%)	9 (90%)	
Citizenship	Malaysian	6 (5.2%)	109 (94.8%)	0.008
	Non-Malaysian	4 (23.5%)	13 (76.5%)	
Ethnicity	Malay	2 (2.4%)	83 (97.6)	0.034
	Chinese	1 (12.5%)	7 (87.5%)	
	Indian	3 (15.8%)	16 (84.2%)	
	Bumiputra Sabah Sarawak	0 (0%)	1 (100%)	
	Others ethnicity	4 (21.1%)	15 (78.9%)	

## Discussion

Typhoid fever is a common foodborne disease in Malaysia. In Klang Valley, typhoid cases were higher in men (*n* = 272) at 55.6% than in women (*n* = 217) at 44.4%. The mean age of typhoid fever cases in Klang Valley was 29.80 years (±17.44). Most of the typhoid cases were detected among patients aged 21 to 30 years. Other studies in Malaysia have shown that children (0–4 years) and young adults (25–29 years) were more susceptible to typhoid than the older age population [7]. The overall trend of typhoid cases in Klang Valley had increased from 2011 (37 cases), 2012 (44 cases) and 2013 (50 cases). There was a sudden spike of cases in 2015 with 98 cases compared with 36 cases in 2014. This sudden increment was associated with an outbreak that involved construction workers where 13.7% (*n* = 70) of cases from this study were contributed by foreigners (non-Malaysian) from India, Indonesia, Bangladesh, Myanmar and Nepal. Notably, the increased number cases of typhoid from 2011 to 2015 was contributed by foreigners, where five typhoid cases were reported among foreigners in 2011 and further increased to 27 typhoid cases in 2015. This finding is in contrast to an outbreak that occurred in Kelantan, where typhoid was associated with contaminated ice and ready-to-eat food distributed by street hawkers in the night market [17]. In Malaysia, particularly in Klang Valley, the water supply is properly monitored and assuredly clean, with good management of the sewer system; however, typhoid infection remains an important public health problem. Most cases in developed countries occur in migrants, travelers and contaminated food chains by food handlers. A study performed in Japan (2015) reported that 54.8% of typhoid cases were reported from people who had traveled to Myanmar; in 2013, the incremental cases were imported cases from Cambodia [18]. The finding from this study also found that ethnicity contributed to 14.4% (*n* = 73) of typhoid cases by foreigners, representing the third-highest rate after Malay (*n* = 264, 52.1%) and Chinese (*n* = 97, 19.1%).

Typhoid fever is endemic in Malaysia where the country still experiences periodic epidemic outbreaks. In Malaysia, typhoid is more common in Kelantan state where multiple outbreaks were recorded in Kelantan in 2001 until 2007 [7, 19], with a major outbreak in 2005. The estimated annual incidence rate in Kelantan state (which has 10 districts) was 37 per 100,000 population [13]. Other studies in Malaysia reported that the incidence rates of typhoid fever in the Federal Territory of Kuala Lumpur for 1996 and 1997 were 3.68 and 3.78 per 100,000 population respectively [20], but lower incidence rates were observed in our study in Klang Valley with 0.58 and 1.42 per 100,000 population in 2011 and 2015, respectively. The annual incidence rate in Malaysia is between 10.2 and 17.9 per 100,000 population [21]. In contrast, Singapore, an urban country, showed a steady decline in cases from 5.9 per 100,000 to 1.2 per 100,000 over 10 years [22]. Similar to Singapore, a study in Thailand reported that the national incidence trend had decreased from 2008 to 2014 with 8.6 per 100,000 population in 2008 and 3 per 100,000 population in 2014 [23].

Being an urban area in Malaysia, the Klang Valley has 12 public health facilities (government hospital) and an estimated 43 private health facilities (private hospital). Of 507 cases, only 198 patients chose to visit government hospitals for treatment and 309 patients had visited private facilities. Most private companies in Malaysia have their own hospital panel for their employees that is usually a private hospital. Thus, most typhoid patients receive treatment at private hospitals rather than at government hospitals. From the observation, most of the typhoid cases (*n* = 184) at government hospitals used the culture method test, which is widely recommended by international society, while private facilities were seen more used to the Typhidot and WWF tests, 110 and 118 cases, respectively, likely because this method was easy to perform and quicker results were produced. According to WHO, the isolation of *Salmonella typhi* from the bone marrow is the current gold standard to confirm typhoid fever cases. However, the equipment, supplies, cost and requirement for personal training render its use limited, particularly in middle- and low-income countries where blood culture is a more practical. However, results from blood culture take up to 3 days, possibly causing delays in treatment. Thus, the rapid test is more widely used in many countries, including Malaysia, to diagnose typhoid fever.

e-Notification, formerly known as Communicable Disease Control Information System (CDCIS), is a web-based application implemented by the Disease Control Division, Ministry of Health Malaysia where only the registered authorized public health officer can access the system. Notifications are received from public health facilities, including health clinics, outpatient departments, and government hospitals, as well as from private hospitals and general medical practitioners [24]. Notification of any suspected, probable or confirmed cases are mandatory under the Prevention and Control of Infectious Diseases Act 1988 within 7 days from the diagnosis date to the nearest district health office, but only laboratory-confirmed cases should be registered. From this study, 78.9% of confirmed cases were reported, indicating that typhoid cases were under-reported in the system and notification was possibly not performed. However, the reporting coverage rates of unconfirmed cases (79.5%) and PVP (89.3%) were high. This finding was much higher than that in a study in Kelantan, which had a reporting coverage of 69%, with 22% unconfirmed cases and 43% PVP [13].

Usually, the antimicrobial susceptibility test will be performed to determine the sensitivity of the drug that can be used for patient management or treatment. The mortality in typhoid fever was reduced after the introduction of the antibiotic drug [3]. This susceptibility test should be conducted following the guidelines from the Clinical and Laboratory Standard Institute (CLSI) or European Committee on Antimicrobial Susceptibility Testing (EUCAST) [25]. As a recommendation by CLSI and EUCAST, *Salmonella typhi* should be tested for its susceptibility to ampicillin, chloramphenicol, co-trimoxazole, ciprofloxacin, ceftriaxone and azithromycin. However, the drug panel can be expanded based on local resistant patterns. Normally, for typhoid fever, patients will be treated using ampicillin, chloramphenicol and co-trimoxazole because these are the first-line antibiotics [14, 19, 26]. Across our study, among the seven antibiotics that were tested, nalidixic acid reported the highest antimicrobial resistance with 36.0%, followed by ampicillin, co-trimoxazole and chloramphenicol at 11.1, 9.8 and 9.5%, respectively. Additionally, 8.1% of *Salmonella typhi* cases were resistant to tetracycline, 6.5% to ciprofloxacin and 1.6% to ceftriaxone. However, the sensitivity to common antibiotics (ampicillin, chloramphenicol and co-trimoxazole) was still high (more than 88%) in Klang Valley. A study conducted in Thailand from 2003 through 2014 showed that most *Salmonella typhi* cases were susceptible to cefotaxime and norfloxacin, and resistance to ampicillin, cefotaxime, norfloxacin and co-trimoxazole remained below 40% [23]. A separate study in Bangladesh reported a prevalence of *Salmonella typhi* isolates expressing high-level of resistance to ciprofloxacin of 90.6% [27], with 43% showing resistance to co-trimoxazole and chloramphenicol, and 40% to ampicillin and nalidixic acid [19]. After the MDR *Salmonella* arose toward first-line antibiotics and became the major problem in several countries worldwide, fluoroquinolones (ciprofloxacin) was chosen for patient treatment and had a high cure rate among the carriers [28–30]. Ceftriaxone, a third-generation cephalosporin, and azithromycin (a macrolide) are now used as other options (second-line antibiotics) to treat typhoid fever when first-line drugs and fluoroquinolone cannot be used [31, 32]. In our study, azithromycin was not a routine treatment for typhoid fever and ceftriaxone was used when necessary. Only the medical doctor can prescribe the antibiotic, and pharmacies will release the antibiotic according to the prescribed form. A case report by Phoon et al. in 2015 in Singapore reported *Salmonella* resistance to azithromycin for the first time to ciprofloxacin and ceftriaxone [28]. The development of resistance is likely due to misuse, overuse and inappropriate prescribing procedures [27, 30].

MDR *Salmonella typhi* strains emerged in Southeast Asia in the 1990s [3]. Of 132 typhoid cases in Klang Valley, 10 (7.3%) were MDR *Salmonella typhi*. The trend of MDR *Salmonella typhi* increased from 10% in 2011 to 30% in 2015. Compared with a study in Egypt (2000), the prevalence of MDR *Salmonella typhi* increased from 19% in 1987 to 100% in 1993, but it subsequently decreased to 5% in 2000 [33]. The increasing pattern of MDR *Salmonella typhi* was also observed in Bangladesh [27], Kenya [34] and Africa [24]. The findings from these studies also showed a significant difference between genders regarding MDR *Salmonella typhi* (*p* = 0.004), but no significant difference was found in age (*p* = 0.735). Compared with a study in Islamabad, Pakistan, no significant difference was found between gender and age group regarding MDR *Salmonella typhi* [14]. Several other studies also reported that the frequency of MDR typhoid fever was higher in men than in women but was higher in children than in adults [30, 32, 35]. This is likely due to young children’s unhygienic habits and their dependence on adults for food, who may be carriers of the MDR strains [30]. A significant difference was also seen in citizenship (*p* = 0.008) and ethnicity (*p* = 0.034) in Klang Valley. A study showed that microbial resistance among Asian travelers was the highest. The resistance rate for travelers from India, Pakistan and Bangladesh were 75, 80 and 60%, respectively [14].

### Strengths and limitations

This study provides data on the burden of typhoid fever in Klang Valley, Malaysia that included data from unreported typhoid fever cases. Typically, most reports will utilize data from the national surveillance system, which tends to miss data underreported by private practitioners. This study also described the trends of typhoid cases/incidences in Klang valley and a pattern of antibiotic resistance and provided data on the MDR strain.

However, this study possessed limitations. The source of data from this study came from multiple sources; hence, the data we received were not in the standard variable list required from them. For the laboratory tests, private hospitals and laboratories have their own regulations and laboratory procedures for typhoid fever testing. Various testing methods (Culture, Typhidot and WWF) were performed for typhoid diagnosis or screening. Some health facilities or laboratories only performed the Typhidot and WWF test for typhoid fever without confirmation with the culture test, and no data on additional identification methods were stated for the culture test. We also had very limited information or data on the antibiotics susceptibility results, which were not available in the e-Notifikasi system. Data analysis for antibiotics susceptibility was performed based on the data obtained by several health facilities (*n* = 132), but not all hospitals and laboratories tested their samples with the full panel of antibiotics suggested and no data on the antibiotic concentration were received.

## Conclusion

Local transmission of typhoid fever is still prevalent in the Klang Valley despite rapid urbanization and development in recent years. The incidence of typhoid fever in Klang Valley increased over the last 5 years but remains low compared with that in other Asian countries. The coverage reporting for confirmed typhoid cases in Klang Valley was considered high (78.9%), and this finding is critical for the public health practitioners to prevent typhoid in urban areas. Additionally, antimicrobial resistance among cases is increasing over the years. Good practice in antibiotics use should continue and awareness should be given to patients about the importance of taking antibiotics during the period that will control drug resistance toward the microorganism. These findings are essential for policy makers to plan and implement focused and effective preventative activities to curb typhoid infection in urban areas. The cost of typhoid fever treatment is high every year. Therefore, the introduction of new interventions such as a typhoid vaccine will be a demand in developing countries [36]. The policy should call for compulsory vaccine immunization for food handlers and vendors and also recommended immunization for individuals traveling to highly endemic areas before coming to Malaysia.

## Data Availability

This study used secondary data from the electronic system approved by health institutions and data collection from government and private laboratories. The datasets used and/or analyzed during this study are included in this published article and available from the corresponding author on reasonable request.

## Abbreviations

MDR: Multidrug-resistant
PVP: Positive predictive value
WWF: Widal Weil Felix
DALYs: Disability-adjusted life years
CDCIS: Communicable Disease Control Information System
WHO: World Health Organization
CLSI: Clinical and Laboratory Standard Institute
EUCAST: European Committee on Antimicrobial Susceptibility Testing
